# Medical Appointments

**Published:** 1854-07

**Authors:** 


### MEDICAL APPOINTMENTS.

Our friend, Dr. John Dawson, editor of the *Ohio Medical and Surgical Journal*, has been elected professor of Anatomy and Physiology in Starling Medical College, at Columbus. Dr. Dawson is one of the most industrious men in our profession. He has been working hard all his life, and by persevering study has made himself an accomplished physician. We rejoice in his success, and feel that we venture nothing when we predict, that he will fill worthily and acceptably the chair to which he has been called by the Trustees of Starling Medical College.

We record, with pleasure, the appointment of another worthy member of the profession to a place in another medical college. John A. Warder, M. D., has been elected to the chair of Chemistry in the Medical College of Ohio, long adorned by the talents of Locke. Dr. Warder has a decided taste for natural history, and will delight in the researches of the laboratory. We hope that he and his colleagues may be successful in giving stability to the organization of this college, which has been so long a theatre of revolution and change.

In the Medical College of Virginia, at Richmond, Dr. Beverly R. Wellford, late President of the American Medical Association, has been selected to fill the chair of Materia Medica, and Dr. Brown Sequard as professor of the Institutes of Medicine. The "Mother of States" seems to be waking up to her interests in regard to medical teaching, and to be in earnest about sustaining her medical college at Richmond. The appointments just mentioned are as good as could have been made.

We regret to learn that our esteemed friend, Prof. Bartlett, has not yet so far recovered his health as to be able to discharge his duties in the College of Physicians and Surgeons of New York. The course on Materia Medica will be given, next session, by Prof. Smith, the course on Practice by Prof. Alonzo Clark, and the course on Physiology by Prof. Dalton. Prof. Dalton is comparatively a young man yet, but he is acquiring great distinction as an original investigator, and as a lecturer on medicine we have no superior in our country to Prof. CLARK. The College of Physicians and Surgeons of New York is venerable for age, and most respectable for the ability and dignity of its corps of professors.

We find the following notice in the last number of the *Medical Examiner.* We have the pleasure of knowing Dr. STILLE personally, and heartily endorse every word of the editor in respect to him:—

MEDICAL DEPARTMENT OF PENNSYLVANIA COLLEGE.—We learn, with much pleasure, that the Trustees of this Institution have lately appointed Drs. Alfred Stillé and John Neill to the Chairs of Practice of Medicine and Surgery. These selections are eminently judicious, and must greatly strengthen the School. Dr. Stillé is one of the physicians of St. Joseph's Hospital, of this city, and was for many years Lecturer on the Practice of Medicine in the Philadelphia Association for Medical Instruction, in both of which positions he has acquired a high reputation as a sound and eloquent teacher. Dr. Stillé is also well known to the profession as a frequent contributor to our medical literature. His work on Pathology is highly esteemed, and his report on Medical Literature, presented some years ago to the American Medical Association, will long be remembered as one of the most brilliant papers ever recorded in its Transactions.

Dr. Neill, we think we may safely say, combines many qualifications for the responsible chair which he has accepted. He was for several years Demonstrator of Anatomy in the University of Pennsylvania, and there established a reputation as one of the best anatomists and lecturers in our country. For some years he has occupied the situation of Surgeon to the Pennsylvania Hospital, and has delivered several courses on Clinical Surgery. From his position in this great school of Surgery, and from his long experience as a lecturer both on Anatomy and Surgery, Dr. Neill's success as a Lecturer on the Principles and Practice of Surgery may be confidently predicted.

In the University of Edinburgh PROF. FORBES, one of the most zealous and eminent naturalists of the age, has been elected to the Chair of Natural History, long filled by PROF JAMESON.

